# Protocol: an improved method to quantify activation of systemic acquired resistance (SAR)

**DOI:** 10.1186/s13007-019-0400-5

**Published:** 2019-02-14

**Authors:** José S. Rufián, Javier Rueda-Blanco, Carmen R. Beuzón, Javier Ruiz-Albert

**Affiliations:** 1 Dpto. Biología Celular, Genética y Fisiología, Instituto de Hortofruticultura Subtropical y Mediterránea, Universidad de Málaga-Consejo Superior de Investigaciones Científicas (IHSM-UMA-CSIC), Campus de Teatinos, 29071 Málaga, Spain; 2 Present Address: Shanghai Center for Plant Stress Biology, CAS Center for Excellence in Molecular Plant Sciences, Shanghai Institutes of Biological Sciences, Chinese Academy of Sciences, Shanghai, 201602 China

**Keywords:** Systemic acquired resistance (SAR), Plant immunity, *Pseudomonas syringae*

## Abstract

**Background:**

Plant responses triggered upon detection of an invading pathogen include the generation of a number of mobile signals that travel to distant tissues and determine an increased resistance in distal, uninfected tissues, a defense response known as systemic acquired resistance (SAR). The more direct means of measuring activation of SAR by a primary local infection is the quantification of pathogen multiplication in distal, systemic sites of secondary infection. However, while such assay provides a biologically relevant quantification of SAR, it is hampered by experimental variation, requiring many repetitions for reliable results.

**Results:**

We propose a modification of the SAR assay based on the *Arabidopsis*–*Pseudomonas syringae* pathosystem exploiting the knowledge of source-sink relationships (orthostichies), known to centralize SAR-competency to upper leaves in the orthostichy of a lower primary infected leaf. Although many sources of variation such as genotypes of plant and pathogen, inoculation procedure, or environmental conditions are already taken into account to improve the performance of SAR assays, a strict leaf selection based on source-sink relationships is not usually implemented. We show how enacting this latter factor considerably improves data reliability, reducing the number of experimental repetitions for results.

**Conclusions:**

Direct selection of leaves for both primary and secondary inoculation exclusively within the orthostichy of the primary infected leaf is a key element on reducing the number of experimental repetitions required for statistically relevant SAR activation results.

## Introduction

Systemic acquired resistance (SAR) is a plant defense response triggered by an initial, local infection, which results in increased resistance to virulent pathogens in distal, uninfected systemic tissues. SAR implies the generation, in the primary site of infection, of a number of mobile signals that travel to distant tissues, mainly via the phloem (reviewed in [1–5]).

The efficiency of SAR induction by a primary local infection can be estimated indirectly, by the collection of petiole exudates and detection of the various potential SAR mobile signals in phloem sap (reviewed in [6]), or calculating by Western blot or RT-*q*PCR the relative level of induction of conventional defense marker proteins, such as PR (pathogenesis-related) proteins, in distal tissues [7]. However, the more direct, biologically meaningful measurement of SAR by a primary local infection remains the quantification of pathogen replication in distal, systemic sites of secondary infection.

The analysis of SAR induction by bacterial infection is frequently performed on the well defined, model pathosystem comprised of *Arabidopsis* and *Pseudomonas syringae* pv. *tomato* (Pto), model strain DC3000 [8–12], although alternative models are also occasionally employed [13, 14]. The SAR assays based on the *Arabidopsis*–Pto pathosystem usually employs for the primary (local) infection a Pto strain that behaves as an avirulent strain due to the expression from a plasmid of an heterologous effector (e.g. AvrRpt2 or AvrRpm1), which triggers ETI in *Arabidopsis*, although inoculation with the virulent strain (Pto DC3000) has also been shown to induce SAR [15]. For the secondary infection on distal leaves a fully virulent strain (Pto DC3000 in this particular pathosystem) is typically inoculated 3 days after the primary infection. Both primary and secondary inoculations are usually performed by pressure infiltration into the apoplast of the leaf tissue, in order to reduce the somehow stronger experimental variation associated with spray inoculations. Bacterial numbers *in planta* at the sites of secondary infection are quantified by tissue maceration and dilution plating, providing a measurement of bacterial replication that constitutes the main output of these assays.

While this experimental approach can provide a measurement of SAR that is direct and biologically relevant, it is in many occasions hampered by considerable experimental variation, which forces researchers to combine a high number of independent experiments to obtain reliable results [9, 12, 16]. Many variables might affect the outcome of such an experiment, among them the genotypes of plant and pathogen, or the environmental conditions. However, genotypes are clearly defined in the *Arabidopsis*–Pto pathosystem, unlike alternative systems using less characterized plants (e.g. *Cucumis sativus*) or bacterial strains (e.g. *P. syringae* pv *syringae* strain D2 or pv *maculicola* strain ES4326) [13, 14, 17]. The same applies to developmental plant stage, bacterial growth conditions, inoculation dose, or infiltration methods, all of which are fairly standard. Environmental conditions (e.g. temperature, irradiation, circadian rhythm), which seem to affect considerably SAR induction [12, 18], can also be closely monitored to reduce variation.

However, there is an additional source of variation that is not regularly addressed when performing SAR assays. As mentioned above, SAR requires that a number of mobile signals travel to distant tissues, something that happens mainly via the phloem although cell-to-cell movement might also contribute [19, 20]. In *Arabidopsis*, source-sink relationships (orthostichies) have been long taken into account in relation to SAR [19], showing that SAR-competency is mainly restricted to upper leaves in the orthostichy (line passing through the bases of leaves situated directly above one another on an axis) of a lower, primary infected leaf, as a consequence of the transmission of SAR signals via the phloem, although some degree of competency is also achieved by leaves outside the orthostichy.

Here we propose that the selection of leaves exclusively in the orthostichy of the primary infected leaf for the purpose of the secondary infection and subsequent analysis of SAR competency, strongly reduces the variability observed in this type of experiment. We have applied successfully such selection in the *Arabidopsis*–Pto pathosystem, not only for the analysis of SAR induction but also for the suppression of SAR by bacterial effectors, an experimental approach fraught with potential experimental variation [15, 21]. This is a simple modification to the protocol that considerably improves the reliability of the generated data, reducing the number of experimental repetitions required.

## Materials

### Reagents and solutions

- Lysogenic Broth (LB) [22] (see REAGENT SETUP)
- Tryptone (Biolife, cat. no. 412290)
- Yeast Extract (Panreac cat. no. 403687)
- Sodium chloride (Panreac cat. no. 121659)
- Bacteriological agar (Panreac cat. no. 402302)
- Sterile deionised water
- Kanamycin (Sigma, cat. no. K-4378)
- Cicloheximide (Sigma, cat. no. C-7698)
- Magnesium chloride (Panreac cat. no. 13139)
- Soil mixture suitable for growing *Arabidopsis* plants

### Equipment

- Growth chambers or controlled environment rooms under short-day conditions (8 h light, 16 h darkness), at 23 °C and 100–150 mE m^−2^ s^−1^.
- 28 °C Incubator
- Petri dishes
- Autoclave
- Spectrophotometer and cuvettes
- 1 ml needleless syringe
- Cork-borer set (Sigma cat. no. *Z165220*)
- Polypropylene Pestles for 1.5 ml microcentrifuge tubes (Sigma, cat. no. *Z359947*) (OPTION 1)
- 2 ml Deepwell Plates (Eppendorf, cat. no. 0030501217) (OPTION 2)
- Deepwell mat (Eppendorf, cat. no. 0030127552) (OPTION 2)
- Generic metal beads (OPTION 2)
- TissueLyser II (Quiagen, cat. no. 85300) (OPTION 2)

### Reagent setup

- Lysogenic Broth (LB): Measure 10 g of Tryptone, 5 g of Yeast Extract and 5 g of NaCl and resuspend into 800 ml of distilled water. Fill up to 1L with distilled water using a measuring cylinder. Add 16 g of bacteriological agar to 1L. Autoclave at 121 °C for 20 min. Cool down to a temperature about 50 °C and add the appropriate antibiotic. Pour about 20 ml of LB agar per 10 cm Petri dish.
- Antibiotics: For *P. syringae* strains carrying plasmids: kanamycin (15 µg/ml). To avoid fungi contamination: cycloheximide (2 µg/ml).
- Magnesium chloride 10 mM: From a 1M stock, add 1 ml to 99 ml of distilled water.

### Protocol

To induce SAR in *Arabidopsis* Col-0, use wild type Pto DC3000, or a derivative carrying an avirulent effector (e.g. AvrRpt2, AvrRm1, AvrRps4). Bacterial stocks and suspensions must be handle in sterile conditions to avoid contaminations. In this protocol, we use both the DC3000 strain and DC3000 expressing AvrRpt2 from a plasmid [23].

### Plant growth

Critical step: Results can be misleading when using plants that do not have the appropriate age or that are affected by different stresses. Try to avoid plants that have unexpected morphological phenotypes or that seem affected by contaminating pathogens. Plant growth and watering conditions may need to be set up in advance.

1. Sow *Arabidopsis thaliana* seeds in the appropriate soil mixture, cover with a transparent plastic lid and stratify during 2 days at 4 °C in the dark, or stratify them before sowing.
2. Move pots to a controlled environment room to grow at 22 °C under short-day conditions (8 h of light and 16 h of darkness), keeping the lid on. Check every 2 days and water the plants slightly if needed, to ensure that the soil is humid, while carefully avoiding excess water in the tray.
3. Two weeks after sowing transplant *Arabidopsis* seedlings into individual pots or tray wells and cover again with a transparent plastic lid during 1 week to minimize stress after the transplanting process.
4. Four-to-five week-old healthy-looking plants are suitable for bacterial inoculations by infiltration.

### Identification of *Arabidopsis* leaves for inoculation

Day 1. Timing: 30 min.

Critical step: Identification of the right leaves is essential to obtain consistent results. It is thus important to train inexperienced researchers for proper leaf identification. When doing so in our laboratory, we have found useful to have the researcher follow the growth of the plants from the appearance of the first true leaves and numbering each leaf as they appear, until they get used to identifying leaves at later stages.

1. Identify cotyledons and leaves 1 and 2 as the first opposing leaves. From leaf 3 forward, leaves sprout in a roughly 130° angle from each other. Leaves 3, 4, 5 and 6 are progressively larger and rounded (Fig. 1). Half of *Arabidopsis* plants grow clockwise (not shown) and the other half counterclockwise (as seen in Fig. 1). It is important to determine the direction of growth in each individual plant for correct leaf identification.

**Fig. 1:**
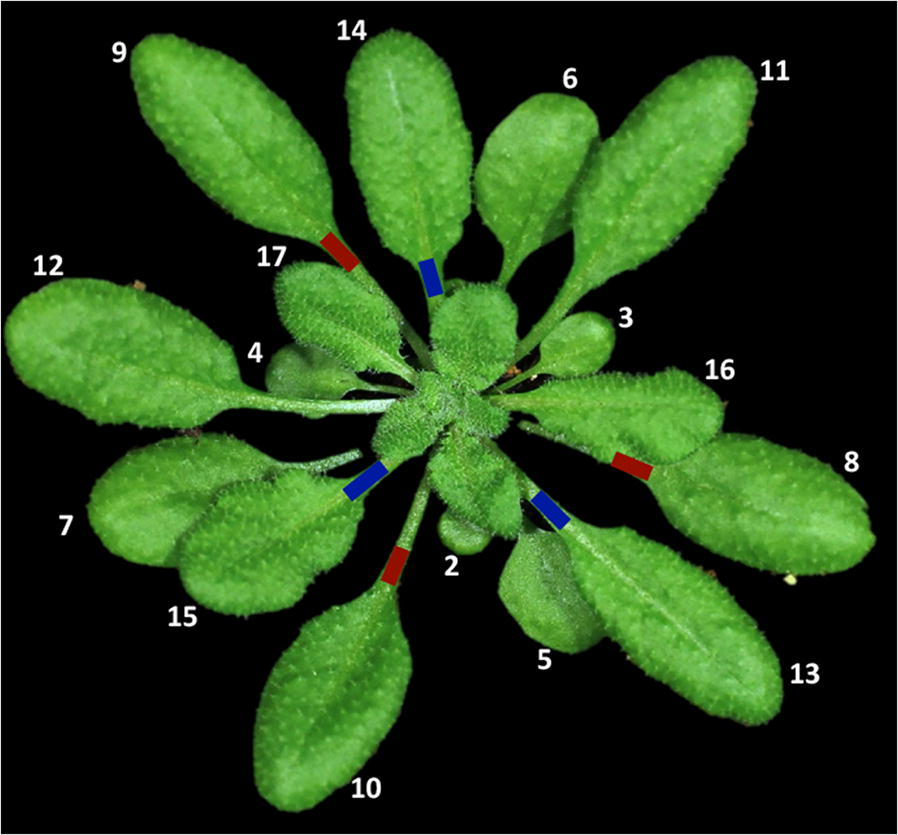
Typical leaf distribution on a 5 week-old *Arabidopsis* plant. The leaves marked in red are used for primary inoculation, while those marked in blue are used for secondary inoculation
2. From leaf 7 onwards, size of the fully expanded leaves is similar and all have a typical elongated shape. To identify the rest of the leaves, follow their distribution at an angle of 130°.
3. Label the petiole of leaves 8, 9 and 10 with a permanent marker pen. Use a different color to label the petiole of leaves 13, 14 and 15 (Fig. 1). This will allow quick identification of the leaves on further steps.

### Preparation of bacterial inocula

Day 1. Timing: 30 min to 1 h.

1. Under sterile conditions, streak out the *P. syringae* strains from a − 80 °C stock culture onto a LB agar medium plate supplemented with the appropriate antibiotics. When using strains carrying plasmids, it is advisable to culture bacteria in the presence of the corresponding antibiotic to ensure plasmid maintenance, and incubate 2 days at 28 °C (Fig. 2).

**Fig. 2:**
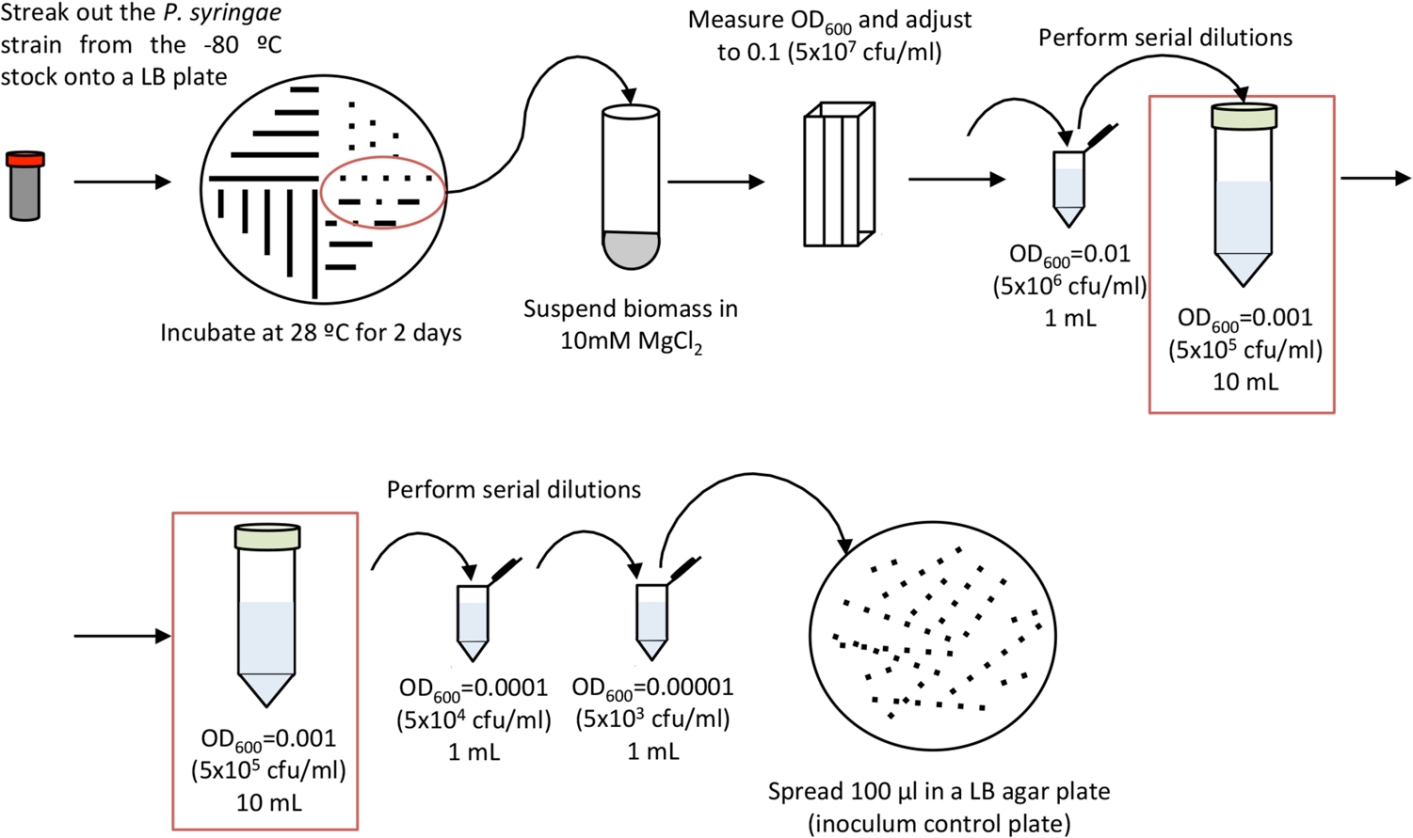
Schematic representation of *P. syringae* inoculum preparation
2. Scrape out bacterial biomass from a fresh Petri dish and suspend in 10 mM MgCl_2_. Adjust the OD_600_ to 0.1 by adding 10 mM MgCl_2_ as needed and confirm OD_600_ using a spectrophotometer. It is extremely important not to overgrow bacteria in the plate, and to avoid 4 °C storage, otherwise the accumulation of extracellular compounds may alter the OD_600_). An OD_600_ of 0.1 in a bacterial suspension of *P. syringae* corresponds to approximately 5 × 10^7^ cfu (colony forming units)/ml.
3. Perform serial dilutions by adding 100 µl of the mixed inoculum to 900 µl of 10 mM MgCl_2_ in a sterile 1.5 ml tube and mix by vortex. Repeat this step to obtain a bacterial suspension containing approximately 5 × 10^5^ cfu/ml (OD_600_ = 0.001).
4. Before proceeding to plant inoculation, collect an aliquot of the 5 × 10^5^ cfu/ml inoculum and make 2 additional serial dilutions (1:10 and 1:100 to a final concentration of 5 × 10^3^ cfu/ml). Plate dilutions in LB agar plates, incubate these plates at 28 °C, and use the resulting colony counts as inoculum control.

### Primary inoculation for SAR activation

Day 1. Timing: 15 min.

Critical Step: To reduce circadian clock variations, perform the inoculations during the first hour of the light cycle.

1. Use 3 *Arabidopsis* plants for each treatment. This method reduces variability between samples, thus 3 plants per biological sample are sufficient for consistent results.
2. Use a needleless syringe to pressure infiltrate the 5 × 10^5^ cfu/ml inoculum or mock solution (10 mM MgCl_2_) into the abaxial face of leaves 8, 9 and 10 (Fig. 3). Inocula concentrations at this step can be varied but, in order to guarantee activation of SAR, should be maintained higher than those used later for the secondary inoculation (proliferation assay). Carefully press the syringe plunger until the area around the syringe grows darker as the full volume of the suspension enters the leaf. To minimize mechanical damage, the infiltration technique should be rehearsed in advance until mastered appropriately using spare plants and water. *Arabidopsis* leaves that have been appropriately infiltrated should not look any different from non-infiltrated leaves 1–2 h after infiltration. Mechanical damage can distort results.

**Fig. 3:**
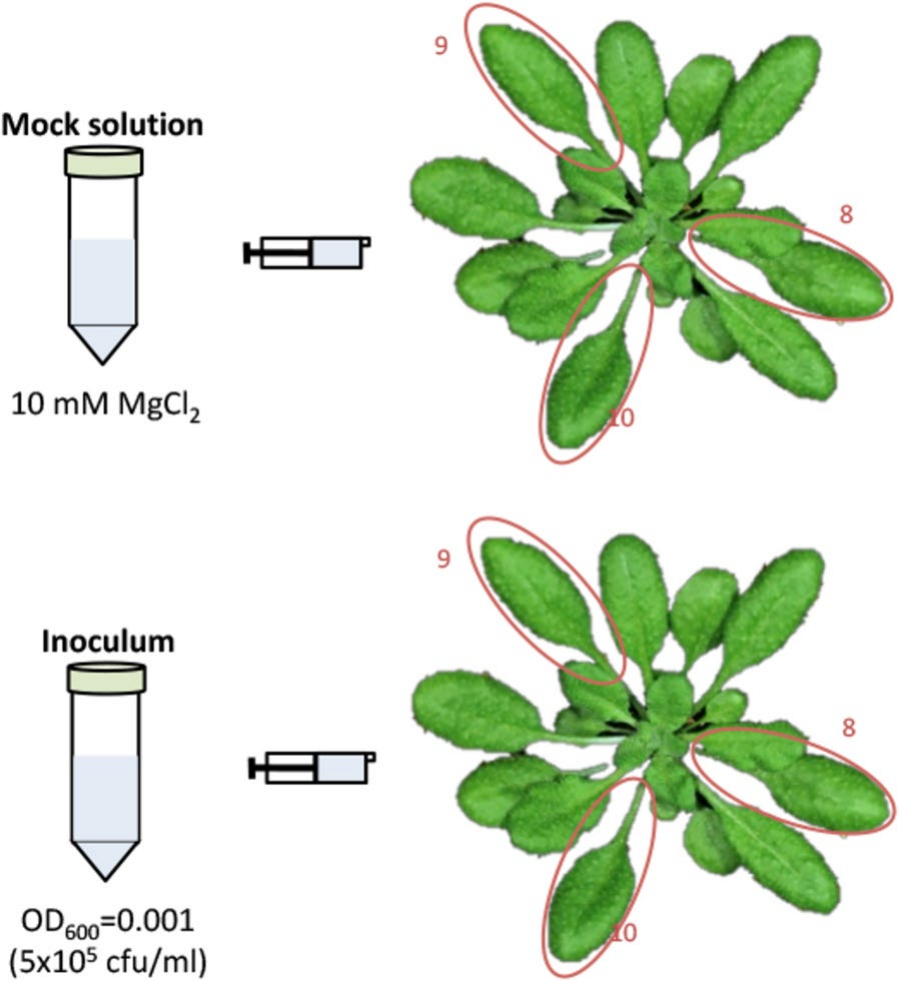
Schematic representation of the primary inoculation process. Inoculum refers to either *P. syringae* pv. tomato DC3000 or to *P. syringae* pv. tomato DC3000 expressing either AvrRpt2 or AvrRpm1 from a plasmid. A minimum of three plants must be inoculated per treatment
3. Infiltrate the three leaves marked for primary inoculation (leaves 8, 9, and 10) and return the plants to the controlled environment room. At this stage, leaves 8, 9 and 10 offer enough leaf area for comfortable infiltration, while also having leaves within their orthostichies (leaves 13, 14 and 15, respectively) that will have equivalently comfortable areas for secondary inoculation.

### Secondary inoculation for SAR determination

Day 3. Timing: 30 min.

1. Prepare a Pto DC3000 suspension following the same steps as in day 1. The inoculation dose must be 5 × 10^4^ cfu/ml (OD_600_ = 0.0001). Plate 100 μl of a further 1:10 dilution (5 × 10^3^ cfu/ml) on a LB agar plate to confirm inoculation dose. This inoculation can be considered a standard proliferation assay, and as such inocula concentration must be low enough to allow for several rounds of bacterial replication leading to differences between treatments that can be reliably detected.
2. Using a needleless syringe, infiltrate the DC3000 5 × 10^4^ cfu/ml suspension into the abaxial face of the leaves 13, 14 and 15 as in day 1 (Fig. 4).

**Fig. 4:**
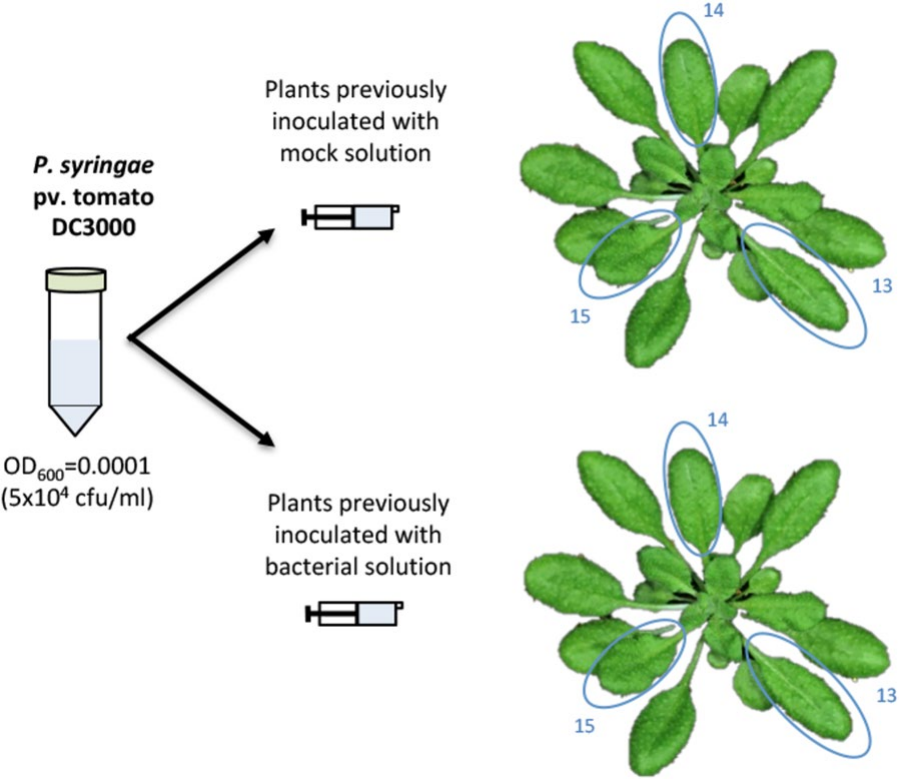
Schematic representation of the secondary inoculation process. A minimum of three plants must be inoculated per treatment
3. Return the plants to the controlled environment room.

### Bacterial recovery from plant samples and cfu determination

Day 7. Timing: 1–2 h

OPTION 1: This option does not require specific equipment and is better suited for small-scale experiments.

1. Four days after secondary inoculation, take one 10 mm-diameter disc using a sterile cork-borer from the center of each of the leaves infiltrated during secondary inoculations (13, 14 and 15). Place it into a sterile 1.5 ml tube containing 500 μl of 10 mM MgCl_2_. Repeat this procedure with each of the inoculated plants (Fig. 5).

**Fig. 5:**
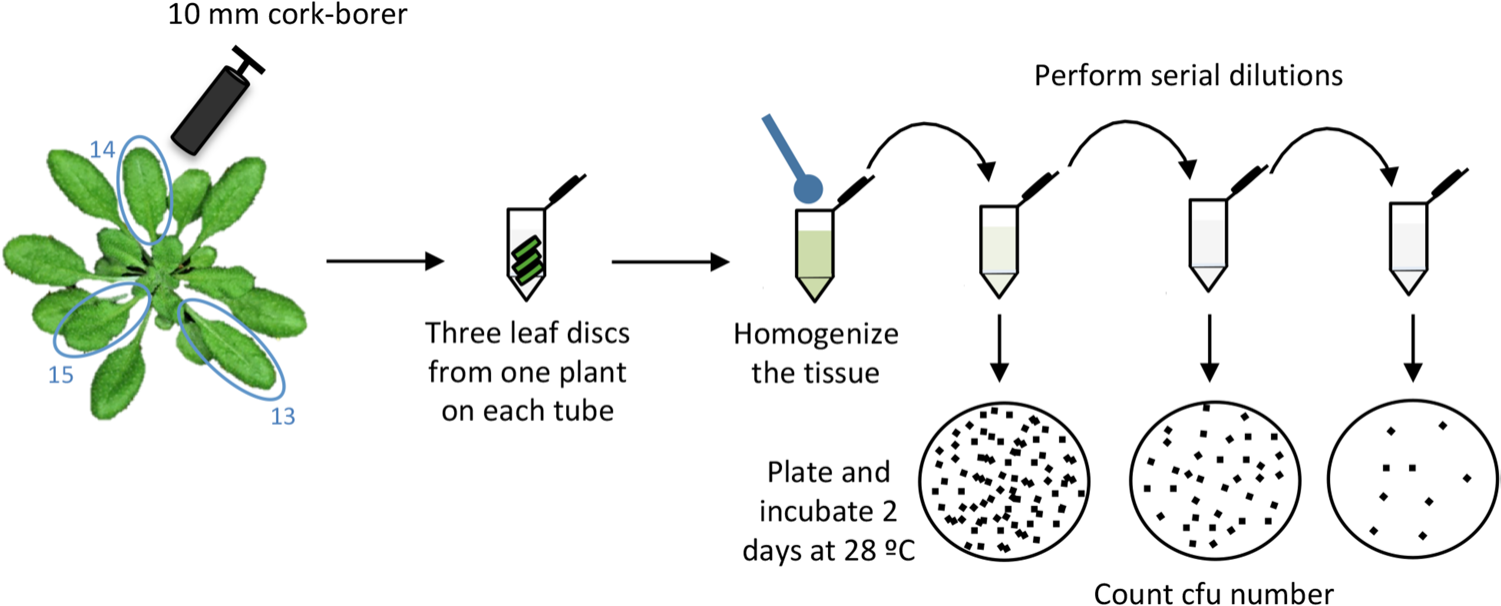
Schematic representation of the bacterial recovery method 1
2. Homogenize plant samples by mechanical disruption using sterile pestles. Add 500 μl of 10 mM MgCl_2_ for a final volume of 1 ml and mix by vortex.
3. Make serial 1:10 dilutions and plate them in LB agar plates containing cycloheximide (2 µg/ml) to avoid fungal contamination. Several serial dilutions should be plated to ensure that enough colonies are available for reliable counting. Depend on the strength of the SAR response triggered by the strain used this will be achieves in one or other dilution. We recommend to prepare each dilution step by adding 100 µl of bacterial suspension into 900 µl of 10 mM MgCl_2_ and to plate 100 µl of each dilution into a 9 cm Petri dish.
4. Incubate the plates at 28 °C for 2 days.

**Fig. 6:**
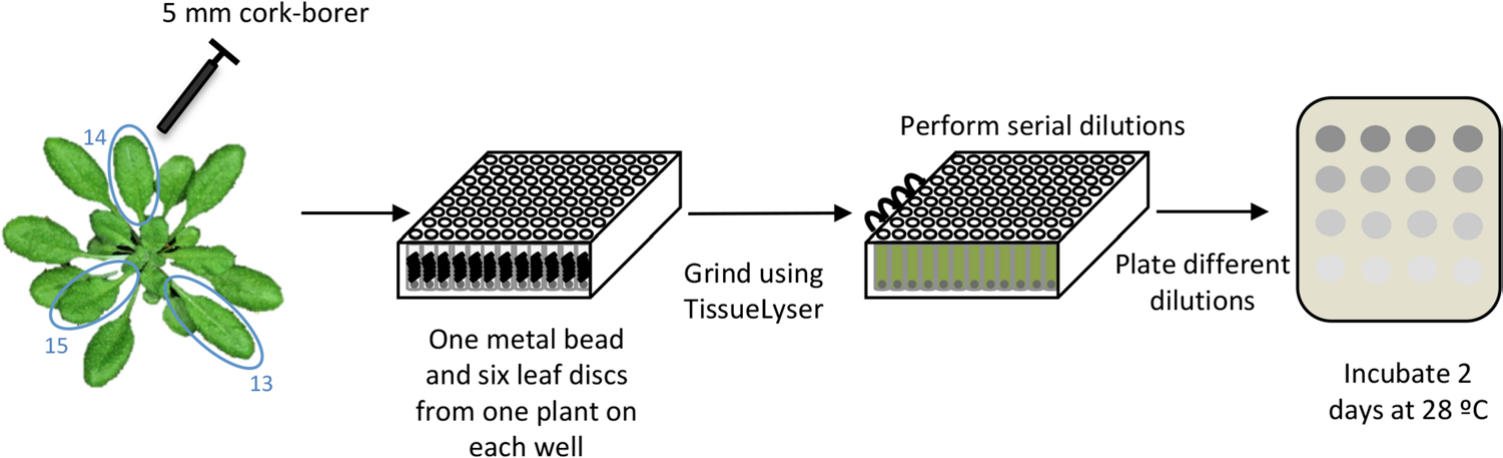
Schematic representation of the bacterial recovery method 2

OPTION 2: We recommend this option for large-scale experiments, since automatized tissue disruption and serial dilution methods described below will save time. However, specific equipment such as a TissueLyser and deep-well plates is required (Fig. 6).

1. Four days after secondary inoculation, take two 5 mm-diameter leaf disc from each infiltrated leaf (13, 14 and 15), one from each side of the center vein of the leaf, using a sterile cork-borer.
2. Place both discs into a well of a 2 ml deep-well plate containing 500 μl of 10 mM MgCl_2_ and a metal bead. Repeat this procedure with each of the inoculated plants, following a line of wells. Cover the plate with a plastic mat and seal using a roller or a 50 ml Falcon tube. Avoid the use of first and last lines and columns, since the mat sometimes does not seal correctly those wells and some liquid could leak.
3. Grind the tissue using a TissueLyser or equivalent. Program one cycle 30 s with a frequency of 30/s. Change the orientation of the plate and repeat the cycle. The smooth adapter plate of the TissueLyser must be placed facing the plastic mat.
4. Spin the plate for 15 s at 4000 rpm to take down any debris that may remain stuck to the mat.
5. With a multi-channel pipette, add 500 μl of 10 mM MgCl_2_ and mix by pipetting up and down.
6. Add 900 µl of 10 mM MgCl_2_ to each well.
7. Using a multi-channel pipette, add 100 µl of the grinded suspension to the next well containing 900 µl of 10 mM MgCl_2_ to perform 1:10 dilution. Mix by pipetting up and down. Check carefully that all the tips take the same volume. Large plant debris could jam the tip. If this happens, just wait for 1 min for large debris to sediment.
8. Plate the dilutions in LB agar plates containing cycloheximide (2 µg/ml) to avoid fungal contamination. Plate 100 µl of each dilution into a 9 cm Petri dish.
9. Incubate these plates at 28 °C for 2 days.

### Quantifying SAR activation

Day 9. Timing: 30 min.

1. Count the number of colonies from each plate containing between 50 and 500 colonies. Multiply this number by the dilution factor to obtain the cfu/ml. Notice that by plating 100 µl on a plate you are further diluting by a tenfold. Thus, the cfu on a plate coming from a 10^−4^ dilution must be multiplied by 10^5^.
2. Divide the cfu/ml value by the leaf area used, in order to express bacterial numbers as cfu/cm^2^. For example, if the 10 mm cork-borer was used, divide by 2.37 (0.79 cm^2^ each disc, multiplied by 3 discs).
3. Calculate the mean and standard deviation for each sample.
4. Use a 2-tailed Student’s t-test and the null hypothesis: mean value of the sample is not significantly different from the mock sample (*P* value < 0.05) to establish whether differences observed are statistically significant.

## Results

**Fig. 7:**
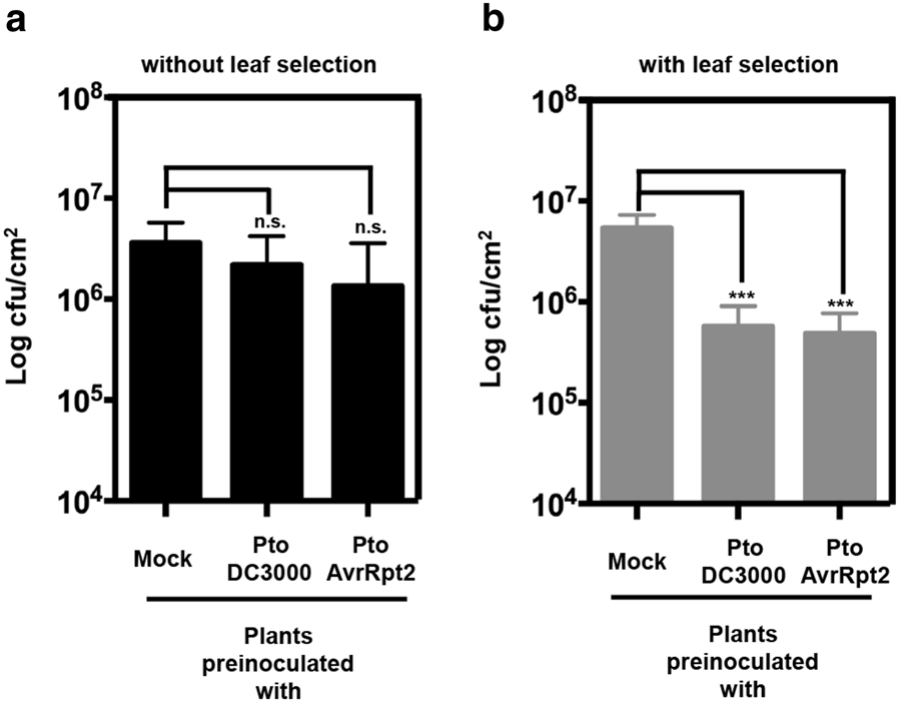
Typical results of bacterial counts in SAR experiments without (**a**) or with (**b**) leaf selection, as described in this protocol. Primary leaves were inoculated with MgCl_2_ (Mock), virulent (Pto DC3000) or avirulent (Pto expressing AvrRpt2) bacteria. Secondary leaves were inoculated with Pto DC3000 and bacteria were recovered as described in option 1. Three *Arabidopsis* plants were used per treatment. Asterisks indicate significant differences compared to the Mock control according to a Student’s t-test (*P *< 0.001)

In our lab, we typically perform the analysis of SAR induction using the model pathosystem comprised of *Arabidopsis* and Pto DC3000 [15, 21], and often use for the primary (local) infection both virulent Pto DC3000, or avirulent Pto DC3000 expressing from a plasmid the heterologous effector AvrRpt2, which triggers ETI in *Arabidopsis*. For the secondary infection on distal leaves we always use fully virulent Pto DC3000, inoculating 3 days after the primary infection. For years we encountered large variation between independent experiments, and also between replicas within the same experiment. Figure 7a shows an example of the results we used to obtain when such large variation was encountered prior to the protocol optimization described here. Before optimization, the activation of SAR was most of the times only hinted by a trend in the mean values for Pto DC3000 cfu/cm^2^ obtained from leaves pre-inoculated with Pto DC3000, and more noticeably with Pto DC3000/pAvrRpt2, being lower than those obtained for mock-inoculated leaves, however such differences were seldom statistically significant. As other labs have reported, we went through careful checking and normalization of the many variables that seemed to influence the outcome of the experiment, paying particular attention to those seemingly reducing variation [9, 12, 16]. We made the observation that the experiments carried out by a particular researcher from the lab, who always inoculated leaves in the same position in all plants, both for primary and secondary inoculation, displayed noticeably smaller variation. Following this rather chancy observation we looked into whether taking into account source-sink relationships (orthostichies) [19] for our experimental design could be behind the reduced variation observed. Thus we got to the experimental setup described in this protocol. Of all the variables tested throughout our struggle to reduce variation, this single refinement alone provided by far the strongest reduction of experimental variation, so currently we can regularly obtain reliable results even with the use a comparably small number of replicas and independent experiments. Figure 7b shows results from a typical experiment carried out using the optimized protocol hereby presented. Mean values of Pto DC3000 cfu/cm^2^ obtained from leaves pre-inoculated with either Pto DC3000, or Pto DC3000/pAvrRpt2 display a consistent tenfold reduction compared to mean values obtained for leaves mock-inoculated, and variation is consistently smaller than obtained using the previous experimental setup, and statistically significant. We have not tested whether leaf selection would also improve reproducibility reducing variation for other SAR models. However, since it pivots on the plant host, we expect that any SAR assays carried out in *Arabidopsis* using other pathogenic microorganisms should benefit similarly. For SAR models based on different plant hosts, leaf selection would have to be optimized, however the information that secondary inoculation should take place in the orthostichies of the primary infected leaves could still be helpful.
